# Potential of combined red and near-infrared photobiomodulation to mitigate pro-osteoclastic and inflammatory gene expression in human mandibular osteogenic cells

**DOI:** 10.1007/s10103-024-04180-2

**Published:** 2024-10-01

**Authors:** Biagio Palmisano, Alessandro Del Vecchio, Alfredo Passaretti, Alessia Stefano, Giovanna Miracolo, Giorgia Farinacci, Alessandro Corsi, Mara Riminucci, Umberto Romeo, Andrea Cicconetti

**Affiliations:** 1 Department of Molecular Medicine, Sapienza University of Rome, Viale Regina Elena 324, 00161 Rome, Italy; 2 Department of Oral Maxillo-Facial Sciences, Sapienza University of Rome, Via Caserta 6, 00161 Rome, Italy

**Keywords:** Osteoprogenitors, Mesenchymal stem cells (MSCs), Bone remodeling, Osteoclastogenesis, Low-level laser therapy (LLLT), Cytokine modulation, Matrix homeostasis, Jawbone regeneration, Maxillofacial healing

## Abstract

Appropriate regeneration of jawbone after dental or surgical procedures relies on the recruitment of osteoprogenitor cells able to differentiate into matrix-producing osteoblasts. In this context, photobiomodulation (PBM) has emerged as promising therapy to improve tissue regeneration and to facilitate wound healing processes. The aim of this study was to determine the effect of PBM on human osteoprogenitor cells isolated from mandibular trabecular bone.

Bone marrow stromal cell cultures were established from 4 donors and induced toward osteogenic differentiation for 14 days in a standard osteogenic assay. Cells were irradiated with a combined red/near-infrared (NIR) laser following different schedules and expression of osteogenic, matrix-related, osteoclastogenic and inflammatory genes was analyzed by quantitative PCR.

Gene expression analysis revealed no overall effects of PBM on osteogenic differentiation. However, a statistically significant reduction was observed in the transcripts of *COL1A1* and *MMP13*, two important genes involved in the bone matrix homeostasis. Most important, PBM significantly downregulated the expression of *RANKL*, *IL6* and *IL1B*, three genes that are involved in both osteoclastogenesis and inflammation.

In conclusion, PBM with a red/NIR laser did not modulate the osteogenic phenotype of mandibular osteoprogenitors but markedly reduced their expression of matrix-related genes and their pro-osteoclastogenic and pro-inflammatory profile.

## Introduction

Dental medicine encompasses many types of surgical intervention that demand efficient tissue regeneration in order to restore oral health. Bone regeneration, in particular, is of utmost importance to re-establish the normal anatomy and function of the oral cavity after procedures such as dental extraction or cyst and neoplastic tissue removal, and to guarantee the stability of dental implant treatments. In all these clinical contexts, appropriate regeneration of maxillary and mandibular bone relies on the recruitment of osteoprogenitor cells able to differentiate into matrix-producing osteoblasts [1]. This process is triggered by the surgical procedure itself and, depending on the size of the defect, may or may be not sufficient to achieve complete healing. Indeed, in small size defects, the newly formed matrix produced by bone cells leads to complete self-repair. In contrast, when large size (critical size) defects are produced, autologous bone grafts or synthetic substitutes are required to refill the void but the function of osteogenic cells is still critical to ensure the adequate integration of the filler with the surrounding bone [2]. Unfortunately, the final outcome for many patients, either self-healing or graft osteointegration, is unsatisfactory due to the presence of conditions that reduce proliferation and differentiation of osteogenic cells or enhance bone resorption. These include, for example, age of the patient, local processes that generate an adverse microenvironment enriched in inflammatory cytokines such as periodontitis or periimplantitis, and systemic conditions such as osteoporosis and diabetes mellitus [3]. Consequently, the development of further strategies that could modulate osteogenic cell function and bone healing in these critical contexts is highly needed.

Photobiomodulation (PBM) is “the mechanism by which non-ionizing optical radiation in the visible and near-infrared spectral range, is absorbed by endogenous chromophores to elicit photophysical and photochemical events at various biological levels without eliciting thermal damage” [4, 5] and its clinical application is commonly referred to as PBM therapy (PBMT).

Most of the available studies on PBM reported beneficial effects of red (wavelengths of ∼ 600–750 nm) and near-infrared (NIR) light irradiation (wavelengths of ∼ 750–1100 nm) within the limits of the so called “therapeutic window”, even though more recently, a wide body of evidence suggests that also the visible wavelengths (violet/blue: ~200–450 nm, green: ~530–550 nm and yellow ∼ 580–590 nm) can induce photo-physical and photochemical effects that modulate biological processes [6]. Although controversy still surrounds the application of PBM in daily clinical practice, due to the uncomplete comprehension of how PBM triggers molecular responses and the lack of consensus about photophysics and radiometric parameters that affect repeatability and reliability [7], the possibility to improve tissue regeneration by enhancing wound healing processes [8] or by stimulating different stem cells populations has opened an interesting and fascinating horizon in light induced bioengineering.

In dental medicine, PBM has been tested in dental extraction, periodontal defects, maxillary cysts and other conditions, in some of which it has been shown to exert anti-inflammatory and analgesic effects and to promote bone healing [9]. This demonstrates that PBM can be an important adjuvant in many oral and maxillofacial procedures, contributing to restore a normal microenvironment and to stimulate the function of bone forming cells.

However, no studies are currently available on the effect of PBM treatment on osteoprogenitor cells isolated from human jaw bones. Furthermore, no well-established and univocal protocols of treatment are reported in literature.

The objective of this study was to investigate the ability of red/NIR irradiation to modulate the behaviour of bone marrow osteoprogenitor cells isolated from samples of human mandible bones and grown in a standard osteogenic in vitro assay. We tested three different schedules of cell irradiation and analysed the effect of each of them on the deposition and mineralization of extracellular matrix and on the expression of genes involved in osteogenic differentiation, matrix remodelling, osteoclastogenesis and inflammation.

## Materials and methods

### Human subjects

Patients with an age between 18 and 80 years with a good general health were considered for the study. Specific exclusion criteria were proven osteometabolic diseases, inflammatory and/or infectious pathologic conditions at the site of surgery, genetic alterations, neoplastic disorders, kidney disease, diabetes, chronic inflammatory diseases and treatment with corticosteroids, bisphosphonates or denosumab.

Four subjects heterogeneous as for age and sex but homogeneous as for anatomical site of intervention and sampling, were selected. Three of them underwent extraction of wisdom teeth whereas the fourth underwent implant fixture insertion (Table 1).

**Table 1:** Patients and surgical sites

Sex	Age (years)	Surgical site	Surgery
Male	31	Mandible, posterior third	Third molar extraction
Male	18	Mandible, posterior third	Third molar extraction
Female	23	Mandible, posterior third	Third molar extraction
Female	71	Mandible, posterior third	Implant fixture insertion

### Collection of samples

For this study, waste fragments of bone tissue released during the osteotomy procedure (functional to the basic surgery) were used. They were used in accordance with the Declaration of Helsinki and its later amendments, and the study was approved by the local Ethical Committee (Rif. 7447 – Prot. 0141/2024). Osteotomy was performed with rotary instruments, under irrigation. Using a surgical excavator, the fragments of bone tissue released during the intervention were collected from the surgical site, placed into a tube containing Modified Essential Medium with α modification (αMEM, Merck, Darmstadt, Germany) supplemented with 1% solution of Penicillin and Streptomycin (P/S) and immediately sent to the laboratory under temperature-controlled conditions.

### Cell culture

To isolate bone marrow stromal cells (BMSCs) and osteoblast precursors, jaw trabecular bone fragments were minced in small pieces and incubated in 10 ml of a 2 mg/ml Collagenase II solution (240 U/mg, Gibco) in Hanks’ Balanced Salt Solution (HBSS) for 45 min at 37 °C on a shaking plate. After digestion, 5 ml of αMEM supplemented with 20% Fetal Bovine Serum (FBS), 1% P/S and 1% L-glutamine (L-gln) (complete growth medium) were added and the resulting suspension was filtered through a 100 μm nylon mesh in order to separate bone fragments from dissociated cells. The filtered suspension was then centrifuged at 1300 rcf (relative centrifugal force) for 5 min. The resulting pellet was resuspended in 10 ml of complete growth medium and cells were plated in a 100 mm-diameter dish. Medium was changed twice a week; when the cells reached 80% of confluency were lifted by adding 0,25% Trypsin for 5 min and plated in 24-well plates at the density of 2⋅10^4^ cells per well. Six wells per plate were used and the remaining wells were filled with a Phenol Red-containing αMEM to shield the cell cultures and to avoid a secondary radiation during the laser treatment.

Cells were induced to osteogenic differentiation by incubation for 14 days with Dulbecco’s modified eagle medium (DMEM, Thermo Fisher Scientific, Waltham, USA) with no Phenol Red, supplemented with 10% FBS, 1% P/S, 1% L-gln, 10^− 8^ M Dexamethasone, 4 mM β-glycerophosphate and 50 μg/ml of Ascorbic Acid (Merck). During osteogenic differentiation, cells underwent laser treatment according to different schedules (Table 2). At the end of differentiation, cells were either collected for gene expression analyses or fixed for 10 min with 4% formaldehyde solution for von Kossa and alkaline phosphatase (ALP) stainings.

### Laser treatment study design

A double diode red (650 nm) and NIR (910 nm) superpulsed laser was used (Lumix 2 diode laser, Fisioline LTD, Verduno CN, Italy). The 650 nm emitting diode was set at 100mW in continuous wave, while the 910 nm emitted at 500mW, at 9 kHz. Each irradiation had a duration of 58 s to release a total amount of 4 Joules/well, chosen according to the settings described by many studies [10]. Three different modes of laser administration were performed (Table 2): a single dose on the 1st day of culture (LT 1); repeated dose on the 1st day of culture and then every other day up to 2 weeks (LT 2); a single dose on the 12th day of culture (LT 3).

In order to expose all cells to the same environmental conditions throughout the study both treated and untreated cultures were kept at room temperature during laser irradiation.

**Table 2:** Schedules used for laser treatments (LT)

Condition	Protocol	Schedule
Control	None	----------
LT 1	One shot	Day 1
LT 2	Alternate	Days 1-3-5-8-10-12
LT 3	One shot	Day 12

### Matrix mineralization assessment

The ability of cells to produce matrix mineralization (mineralized nodules) was analyzed by von Kossa stain. Cells were incubated with 1% Silver Nitrate solution for 20 min under ultraviolet light, washed with distilled water and then incubated with 5% sodium thiosulfate solution for 5 min. Pictures of mineralized nodules were taken with an inverted microscope and quantified with Adobe Photoshop. At least 4 fields from each well were photographed, and at least 3 wells for each experimental point were considered.

### ALP assay

Alkaline phosphatase (ALP) is an important enzyme required to enhance the concentration of inorganic phosphates therefore facilitating mineralization [11]. For this reason, it is used as marker of osteogenic differentiation.

ALP cytochemistry was performed using Sigma Aldrich kit reagents (Merck) as previously described [12]. Briefly, 30 mg of Naphtol AS Phosphate were dissolved in 0,5 mL N, N-dimethylformamide and mixed with 100 mL borate buffer with 100 mg of AS blue BB salt. The solution was added to the wells and incubated for 5–10 min at 37 °C. Pictures from at least 4 fields each well were taken and analyzed by measuring the optical density using ImageJ software.

### qPCR gene expression analysis

Total RNA was isolated using the TRI Reagent^®^ following manufacturer’s instructions (Merck). Reverse transcription was performed by using PrimeScript RT Reagent Kit (Takara, Kusatsu, Japan). cDNA samples were used as templates for quantitative PCR (qPCR) analysis on a 7500 Fast Real-Time PCR System (Applied Biosystem, Waltham, MA, USA), performed using PowerUP Sybr Green (Thermo Fisher Scientific) and specific primers (Table 3) recognizing genes involved in osteogenic differentiation (*CBFA1*,* SP7*,* ALPL*,* BGLAP*), matrix deposition and remodelling (*COL1A1*,* COL3A1*,* COL10A1*,* MMP1*,* MMP8*,* MMP13*), osteoclastogenesis (*TNFSF11*,* TNFRSF11B*,* CSF1*) and inflammation (*IL6*,* IL6R*,* IL1B*). The expression level of each gene was normalized to *GAPDH* expression.

**Table 3:** qPCR primer sequences

Gene	Sequence 5*'* - 3*'*
*ALPL*	F: GCTGTAAGGACATCGCCTACCA R: CCTGGCTTTCTCGTCACTCTCA
*BGLAP*	F: CACCGAGACACCATGAGAGC R: CTGCTTGGACACAAAGGCTGC
*CBFA1*	F: CCCAGTATGAGAGTAGGTGTCC R: GGGTAAGACTGGTCATAGGACC
*COLIA1*	F: GATTCCCTGGACCTAAAGGTGC R: AGCCTCTCCATCTTTGCCAGCA
*COL3A1*	F: TGGTCTGCAAGGAATGCCTGGA R: TCTTTCCCTGGGACACCATCAG
*COLIOA1*	F: GGACTTCCGTAGCCTGGTTT R: AGATGACGGTCCCTCTGGTG
*CSF1*	F: TCAGCAAGAACTGCAACAACAG R: CTGCTAGGGATGGCTTTGGG
*GAPDH*	F: GTCTCCTCTGACTTCAACAGCG R: ACCACCCTGTTGCTGTAGCCAA
*IL1B*	F: CCACAGACCTTCCAGGAGAATG R: GTGCAGTTCAGTGATCGTACAGG
*IL6*	F: GACTGTGCACTTGCTGGTGGAT R: TTCTGCCAGTGCCTCTTTGCTG
*IL6R*	F: GACTGTGCACTTGCTGGTGGAT R: ACTTCCTCACCAAGAGCACAGC
*MMP1*	F: ATGAAGCAGCCCAGATGTGGAG R: TGGTCCACATCTGCTCTTGGCA
*MMP8*	F: CAACCTACTGGACCAAGCACAC R: TGTAGCTGAGGATGCCTTCTCC
*MMP13*	F: GCACTTCCCACAGTGCCTAT R: AGTTCTTCCCTTGATGGCCG
*SP7*	F: GTAGGACTGTAGGACCGGAG R: CTCTCCTCTCTGGAGGTCTGG
*TNFSF11*	F: GCCTTTCAAGGAGCTGTGCAAAA R: GAGCAAAAGGCTGAGCTTCAAGC
*TNFRSF11B*	F: GGTCTCCTGCTAACTCAGAAAGG R: CAGCAAACCTGAAGAATGCCTCC

### Statistical analysis

From each patient, at least 4 technical replicates for each analysis were generated. The mean from the 4 replicates was then used to represent each donor in the sample size. In order to show the general trend of the laser treatments on the cells from each donor, results of each biological replicate are shown as relative to each control, which is therefore reported as 1.

A repeated measure one-way ANOVA, corrected for multiple comparisons using Dunnet method was used to compare the effect of the treatment on gene expression analyses, Von Kossa and ALP staining. In all experiments a *p*-value < 0.05 was considered statistically significant. All graphs and statistical analyses were performed using GraphPad Prism version 9 (GraphPad Software, La Jolla, CA, USA).

## Results

### Characterization of mandibular osteoprogenitor cells

**Fig. 1:**
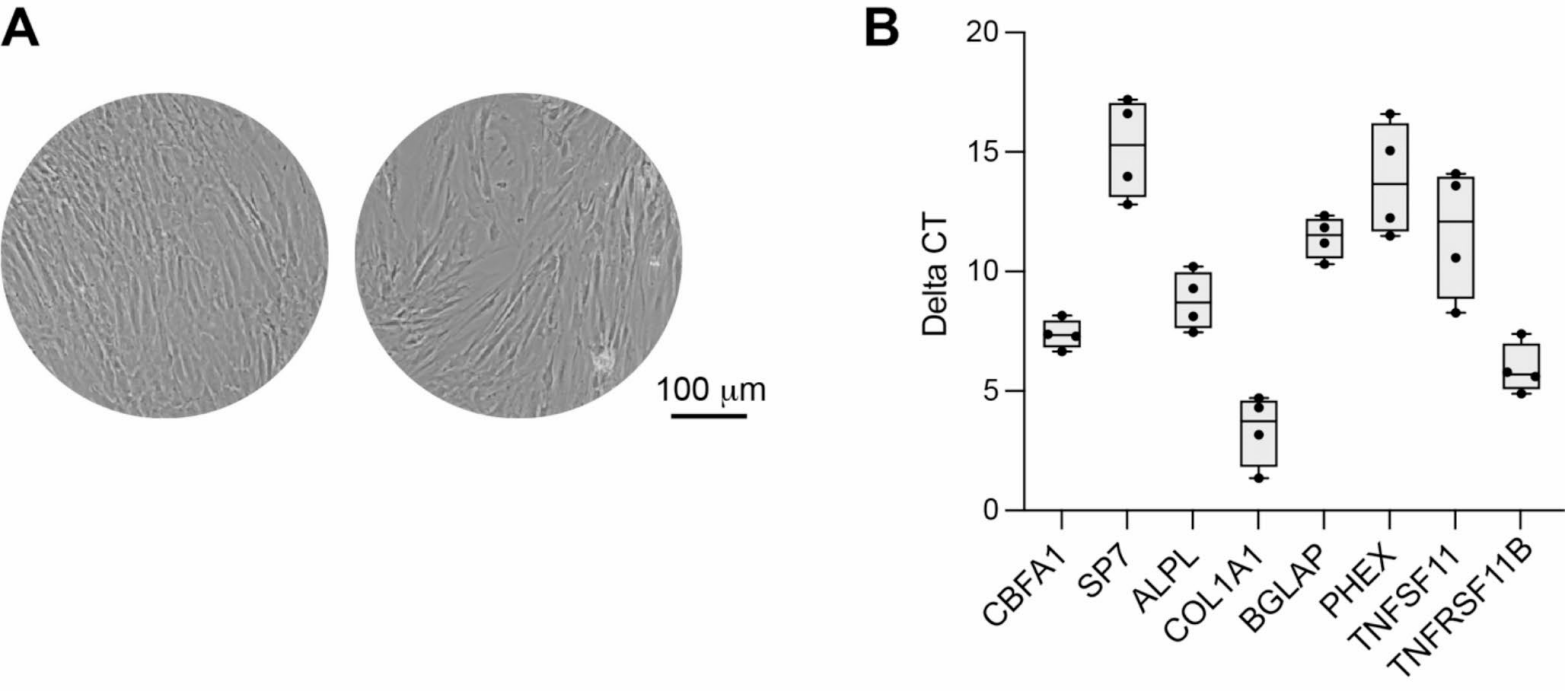
(**A**) Representative pictures of BMSCs isolated from mandibular bone of two patients, after 7 days of expansion in vitro. (**B**) Gene expression analysis by qPCR. The expression of each gene is showed as difference between the CT values of the gene of interest and *GAPDH* as housekeeping gene, so that low values correspond to high gene expression

Cells were isolated from mandible trabecular bone fragments that were recovered as surgical waste from 4 different patients. Cell growth and morphology were overall resembling to those of BMSCs isolated from other canonical skeletal sites, showing an elongated shape and a single nucleus (Fig. 1A). No difference in cell growth and morphology was detected in cultures established from the different patients.

Gene expression analysis was performed after 14 days in a standard osteogenic medium and evaluated as delta CT values between the genes of interest and *GAPDH* that was used as housekeeping gene. This analysis revealed a high expression of *CBFA1* (encoding for RUNX2), a master regulator of the osteogenic commitment and *ALPL*, an enzyme that in bone cells is necessary for matrix mineralization. Moreover, high expression of *COL1A1*, the most abundant collagen in bone, and *TNFRSF11B*, encoding for OPG an important decoy receptor of RANKL was also observed (Fig. 1B). Lower expression of *BGLAP* and *PHEX* were detected, indicating a non-fully mature osteoblast phenotype (Fig. 1B).

### PBM treatment modulates collagen expression but not osteogenesis in mandibular osteogenic cells

**Fig. 2:**
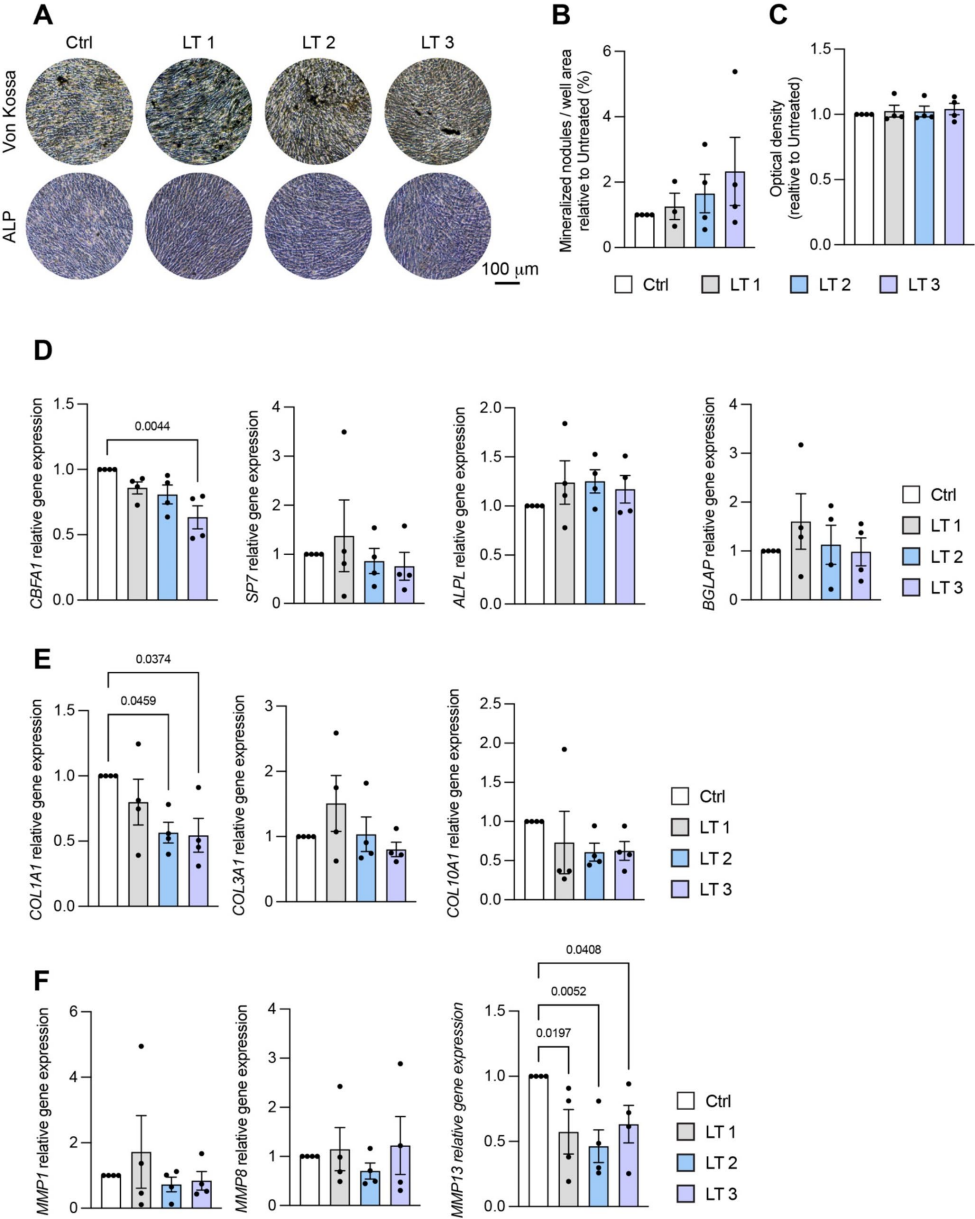
(**A**) Representative images from Von Kossa- and ALP-stained cells cultured for 14 days in osteogenic medium. (**B**) Quantification of the area covered by Von Kossa-stained mineralized nodules. (**C**) Optical density quantification of ALP-stained cells. **D**-**F**) Gene expression analysis performed after 14 days of osteogenic differentiation. For all the genes analyzed, *GAPDH* was used as housekeeping gene. Statistical analysis was performed using One-Way ANOVA test and *p* values < 0.05 from multiple comparisons are reported above each graph

The laser treatment did not affect cell morphology. Moreover, none of the three schedules of treatment significantly modified the ability of the cells to produce extracellular matrix, as revealed by Von Kossa staining of mineralized nodules, albeit a tendency toward an increase compared to non-irradiated samples (control) was observed (Fig. 2A, B). Accordingly, no differences compared with the control were observed in the ALP activity assay (Fig. 2A, C).

On the other side, gene expression analyses revealed interesting effects of the PBM treatment. Overall, the treatment negatively modulated the expression of *CBFA1*, although only LT 3 reduced it in a statistically significant manner (Fig. 2D). No marked effects on the other osteogenic genes were observed with PBM (Fig. 2D), and only slightly higher levels of *ALPL* and Osteocalcin gene (*BGLAP)* were observed in laser-treated cells compared to controls (Fig. 2D). Interestingly, independently of the treatment schedule, in all patients the laser treatment was able to significantly reduce Collagen 1 (*COL1A1)* and Metalloproteinase 13 (*MMP13)* (Fig. 2E, F), two important genes encoding fundamental proteins for bone matrix structure and homeostasis, respectively. Other collagen genes, such as *COL3A1* and *COL10A1* were modulated in three of the four patients, with the former that increased with LT 1 and the latter that decreased with all types of treatments (Fig. 2E).

### PBM treatment reduces RANKL and inflammatory cytokines expression in mandibular osteogenic cells

**Fig. 3:**
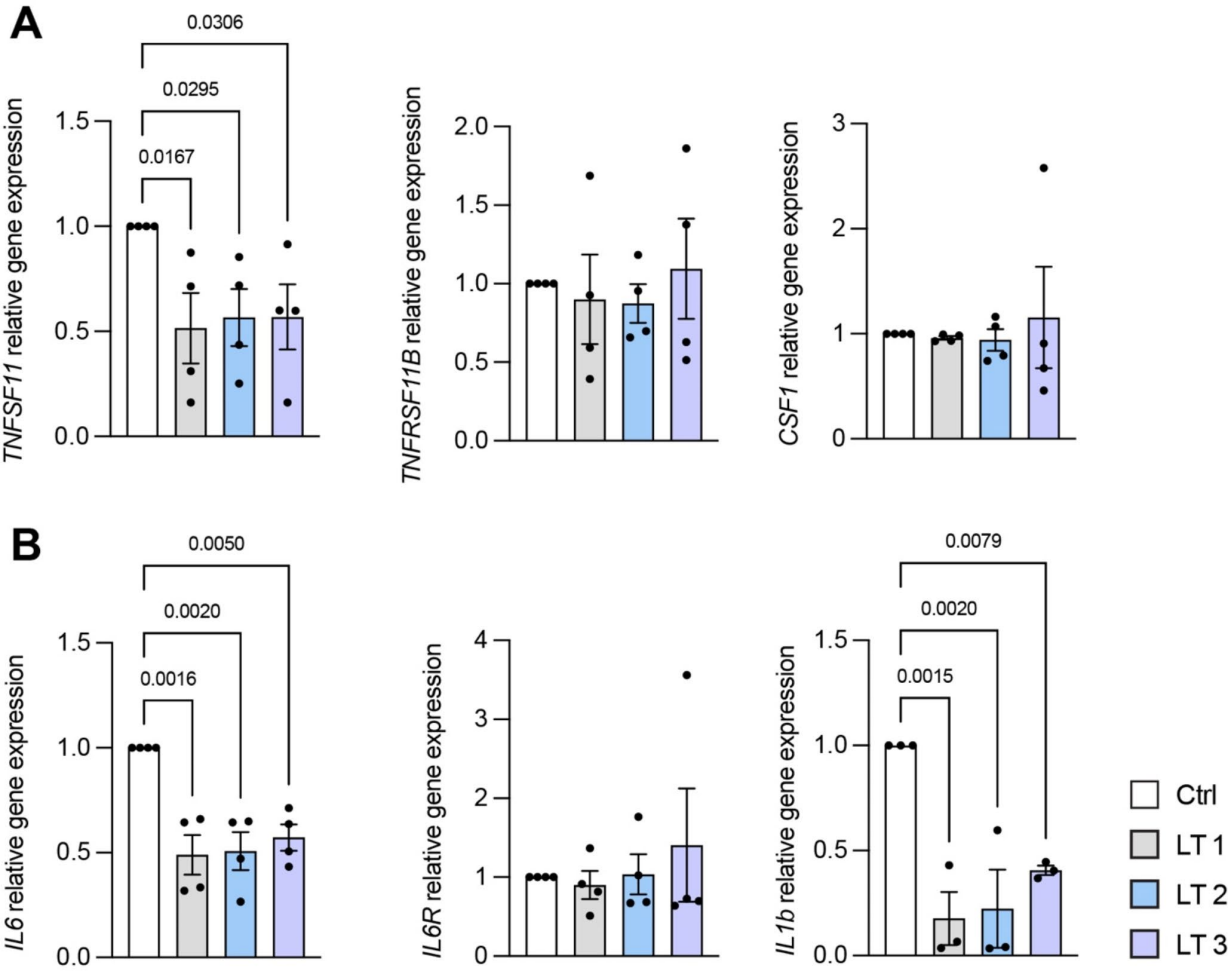
**A**, **B**) Gene expression analysis performed after 14 days of osteogenic differentiation. For all the genes analyzed, *GAPDH* was used as housekeeping gene. Statistical analysis was performed using One-Way ANOVA test and *p* values < 0.05 from multiple comparisons are reported above each graph

We then analyzed the expression of genes involved in the stimulation of osteoclastogenesis and inflammatory response. Surprisingly, we found that all the three different schedules of treatment were able to significantly reduce the expression of *TNFSF11*, encoding for RANKL, the most important osteoclastogenic factor (Fig. 3A). Variable effects of the laser treatments were instead observed on the expression of *TNFRSF11B* (encoding for OPG) compared to control (Fig. 3A), while no effects of the PBM were observed on the expression of the other important osteoclastogenic factor *CSF1* (Fig. 3A).

Interestingly, the expression of molecules involved in the inflammatory response was lowered by laser treatment (Fig. 3B). Specifically, the expression of Interleukin-6 (*IL6)*, one of the most important inflammatory cytokines, which is also known to stimulate bone resorption [13, 14], was significantly reduced by 50% with PBM (Fig. 3B). In addition, a drastic reduction of the Interleukin-1 beta (*IL1B)* transcripts was also observed upon laser treatment compared to untreated cells (Fig. 3B).

## Discussion

The goal of research in all medical fields is to develop novel modalities of intervention able to modulate physio-pathological processes in a faster, safer and less invasive manner compared to available therapies. The PBM has emerged over the years as a treatment able to modulate the activity of different cell types in order to stimulate tissue repair, reduce inflammation and facilitate pain management [15].

PBM collects the legacy of Mester’s studies reporting the stimulatory effect of low-energy radiation on biological systems [16, 17]. Overall, the mechanisms by which lights of particular wavelengths can modulate cellular processes reside in the capability of numerous intracellular elements to transform absorbed light into energy available for specific functions, or to induce the release of discrete amounts of nitric oxide and reactive oxygen species (ROS) that can improve several cell functions at different levels [18].

Recently, some studies started to explore the utility of PBM to improve healing of craniofacial bones with encouraging results. For example, some of them showed that PBM positively affects the post-extraction healing of the socket in different species [19–21] whereas others showed that it improves the osteointegration and stability of dental implants [22, 23]. However, the efficacy of the PBMT is still a controversial matter mainly due to non-homogeneous effects within the different experimental cohorts. A better understanding of the effect of PBM on osteoprogenitor cells that are directly involved in the healing process of jaw bones seems to be critical to clarify this point. Nonetheless, the largest majority of i*n vitro* studies are focused exclusively on osteogenic cells isolated from skeletal samples other than maxillary and mandibular bones, for example from long bones [24], marrow aspirates [25] and cells associated with dental structures such as periodontal cells [26], dental pulp stem cells [27] and cells for exfoliated deciduous teeth [28]. In addition, it is important to remark that most of these studies were performed on animal models rather than on human cells.

It was previously showed that the human maxillary bone hosts a population of osteoprogenitor cells [29] likely involved in bone tissue regeneration following dental surgery. These cells share most of the properties of marrow osteoprogenitors isolated from canonical skeletal sites such as the iliac crest [30] and, most important, are able to form bone in ex-vivo transplants [29].

In this study we analyzed for the first time the effect of a combined red/NIR laser (650 nm and 910 nm) on mandibular osteoprogenitor cells isolated from patients of different ages. We choose a type of irradiation that was previously shown to stimulate cellular processes and bioenergetics, resulting in enhancing wound healing and other beneficial effects [31]. Our results show that the treatment did not overall modify cell growth and osteogenic differentiation, although a single shot of laser treatment two days before the end of the culture interfered with *RUNX2* expression. In our experimental setting, PBM was not able to substantially modulate the expression of *ALPL* or late osteogenic markers such as *BGLAP*, and only a slight increase in the transcripts of these genes was observed in 2 out of 4 patients. Similarly, more mineralized nodules were observed in laser-treated cells compared to controls but only in two out of four samples. Interestingly, PBM seemed to rather decrease the formation and remodeling of the extracellular matrix as suggested by the reduced expression of *COL1A1* and *MMP13* in irradiated cells. Similar effects of PBM are known to occur in other cell types and likely reflect the mechanism underlying the treatment of scars [17]. Interestingly, we observed a clear reduction in the level of transcription of genes involved in inflammation and bone resorption such as *IL6*,* IL1B* and *RANKL*. Previous studies reported that PBM promoted alveolar repair due to the production of growth factors, stimulation of angiogenesis and attenuation of inflammation [32]. Moreover, our results are in agreement with reports on mouse cells demonstrating the anti-osteoclastogenic effect of PBM, by reducing *Rankl* expression [33]. Overall, our data strongly suggest that this effect relies on the reduction of bone resorption and inflammation rather than on the stimulation of osteogenesis. These findings, although very preliminary, open a new perspective for further studies, which must be focused on the effect of PBM on the function of osteoprogenitor cells in promoting tissue remodeling and bone resorption rather than in bone formation. In addition, our results clearly show that no conclusion on the effect of PBM on jaw osteoprogenitor cells may be extrapolated by studies performed on other osteogenic cell populations.

From a clinical perspective, our experiments suggest the possibility to induce a positive biomodulatory effect in the post-surgical healing process, with a combination of wavelengths that can be performant both in the superficial and the deep layers of the surgical bone wound. Furthermore, they provide new useful information to establish a potential protocol of application. This is a particularly controversial aspect, since some studies showed enhanced stimulatory effect on “MSCs” when irradiated with multiple doses [25, 26], whereas other studies [24, 34] reported better results with irradiation performed every other day. Our data show that expression of most genes was modulated independent of the modality of irradiation, suggesting that a single shot might be sufficient to modulate gene expression of mandibular osteoprogenitors, although further experimental and clinical work is required to precisely assess the time schedule for PBMT in patients.
